# Editorial: Case reports in pulmonary medicine 2025

**DOI:** 10.3389/fmed.2026.1927190

**Published:** 2026-07-29

**Authors:** Karolina Henryka Czarnecka-Chrebelska

**Affiliations:** Department of Biomedicine and Genetics, Medical University of Lodz, Łódź, Poland

**Keywords:** diagnostic challenge, innovative therapeutic approaches, pulmonary functionality, rare pulmonary diseases, rare pulmonary mycosis

## Introduction

Respiratory diseases remain among the leading causes of morbidity and mortality worldwide, affecting both the general population and high-risk subgroups (1, 2). Elevated mortality is seen not only in common conditions such as chronic obstructive pulmonary disease and acute lower respiratory infections, but also in severe opportunistic infections, including pulmonary mucormycosis, where reported mortality exceeds 50% (3). In these scenarios, delayed diagnosis, atypical clinical manifestations, or the absence of standardized treatment protocols often contribute to rapid disease progression and poor outcomes. Consequently, case reports and case series are important sources of clinical evidence, helping to recognize rare but potentially life-threatening presentations. This review aims to provide a comprehensive analysis of the predominant themes in case reports published in 2025 in the Frontiers in Pulmonary Medicine Research Topic, with particular emphasis on conditions and complications associated with a high risk of mortality, as well as the implications of these observations for clinical practice.

An analysis of case reports and case series published in 2025 in *Frontiers in Pulmonary Medicine* revealed three dominant thematic areas: (1) rare lung cancers that are difficult to diagnose, (2) severe and atypical pulmonary infections requiring advanced diagnostic and therapeutic methods, and (3) post-operative and perioperative complications in pulmonology and thoracic surgery. Although the individual publications address heterogeneous disease entities, they share a common denominator: a high risk of adverse outcomes, including mortality, as the effect of delayed or inadequate intervention.

## Rare lung cancers—Diagnostic and prognostic implications

Although primary pulmonary lymphoma is rare (< 0.5% of all lung cancers), two case reports published in 2025 contribute to a better understanding of its diagnosis and treatment. Tang et al. describe the case of a 36-year-old woman with an incidentally detected lesion in the lower lobe of the lung, in whom transbronchial biopsy assisted by EBUS-guided TBCB yielded sufficient material to diagnose a follicular splenic lymphoma originating from MALT tissue. The authors highlight the insufficient sensitivity of standard biopsies and the potential of the cryobiopsy technique to obtain adequate tissue samples; they also suggest a “watch-and-wait” approach for asymptomatic stage IV disease (Tang et al.).

The second report (Ni et al.) describes a case of angioimmunoblastic T-cell lymphoma that was initially misdiagnosed as eosinophilic granulomatous vasculitis (EGPA). The use of immunohistochemistry and genetic analyses (TCR gene rearrangement, Epstein–Barr virus RNA *in situ* hybridization) enabled diagnostic correction, which led to a significant change in the treatment strategy (Ni et al.). Both reports emphasize the critical importance of advanced diagnostic techniques—EBUS TBCB, immunophenotyping, and molecular analysis—in cases of suspected primary pulmonary lymphomas. They also highlight the frequent asymptomatic course of the disease and the risk of misdiagnosis.

## Severe lung infections—New technologies in the face of high mortality rates

The second dominant group of reported cases consists of rare and opportunistic pulmonary infections, which are characterized by high mortality, especially in patients with chronic diseases or immunodeficiencies. *Mucormycosis, aspergillosis, atypical mycobacteriosis*, and infections caused by multidrug-resistant bacteria often go undetected using standard microbiological methods. In the reports analyzed, the use of metagenomic sequencing (mNGS), advanced histopathological diagnostics, and non-standard therapies—such as phage therapy—enabled the implementation of life-saving treatment.

One example of an invasive fungal infection, which is often associated with high mortality rates and requires aggressive treatment, is mucormycosis. Zhang and Wang analyzed three cases of pulmonary mucormycosis (a patient with type 1 diabetes; a patient with follicular lymphoma and COVID-19; and a patient with lung cancer and COPD), highlighting that it mainly impacts patients with serious comorbidities like diabetes, cancer, or immunosuppression, and that its progression is frequently severe and can be fatal. In the series described, one patient died despite antifungal treatment, underlining the aggressive nature of the infection. At the same time, the authors demonstrated that multimodal treatment—including pharmacotherapy, bronchoscopic interventions, and surgical treatment—was associated with significantly better outcomes; in the literature review, a survival rate of up to 100% was reported among patients undergoing complex therapeutic management. These results highlight the need for early and aggressive treatment and the importance of integrating various therapeutic methods.

Authors demonstrated that treatment outcomes were strongly associated with the therapeutic approach. In their literature review of 13 previously analyzed cases, mortality was 28.3% among patients receiving antifungal monotherapy and 23.7% among those receiving antifungal therapy combined with bronchoscopic intervention, whereas antifungal therapy combined with surgery was associated with a mortality of only 9%. Notably, all 13 patients treated with a multimodal strategy combining antifungal therapy, bronchoscopic intervention, and surgical management survived (Zhang and Wang).

In turn, Zhu et al. described a case of invasive pulmonary aspergillosis developing secondary to a pleural empyema in a patient with lung cancer, which was hindering the fungal infection symptoms. The diagnosis required the simultaneous use of histopathology and microbiological testing, which confirmed the presence of *Aspergillus fumigatus* and the coexistence of cancer. Treatment included both antifungal therapy and oncological treatment, which led to stabilization of the patient's condition. This case highlights the clinical complexity of opportunistic infections in the context of comorbidities and the need for an interdisciplinary therapeutic approach.

Unlike cases where a fungal infection is present “in the background” of a tumor, Yang and Shu reported a rare instance of pulmonary actinomycosis that resembled a tumor. The infection showed symptoms like chronic hemoptysis and radiographic signs that suggested other illnesses, ultimately leading to a massive, fatal pulmonary hemorrhage. This case emphasizes that even rare fungal invasions can lead to serious complications and may directly result in death. Hence, it is crucial to accurately differentiate pulmonary invasions from tumors.

Similar diagnostic challenges were described in a series of cases of infections caused by the *Mycobacterium avium complex* (MAC), which can mimic pulmonary tumors. Jacobs et al. demonstrated that this infection often leads to a suspicion of lung cancer and, consequently, unnecessary invasive diagnostic procedures, whereas appropriate antibiotic treatment results in regression of the lesions. These cases underscore the importance of proper differential diagnosis and the potential of therapeutic strategies to avoid excessive surgical intervention.

One of the key clinical challenges is the growing resistance to antibiotic treatment. A report by Lin et al. describes a patient with severe pneumonia caused by a highly antibiotic-resistant strain of *Acinetobacter baumannii*, in whom standard therapy remained ineffective for 2 months. The use of a bacteriophage–antibiotic combination therapy led to rapid clinical improvement, confirmed by the elimination of the pathogen and improved respiratory parameters. This case illustrates the potential of alternative strategies, particularly in situations where treatment options are limited, and highlights the role of phage-antibiotic synergy in overcoming microbial resistance.

Analysis of selected case reports reveals that rare and opportunistic lung infections often show high clinical variability and pose a substantial risk of misdiagnosis and poor outcomes. They frequently present with non-specific symptoms and limited usefulness of traditional serological markers. The presented case reports emphasize that employing advanced diagnostic techniques and unconventional treatment approaches is essential for enhancing patient prognosis.

## Postoperative and perioperative complications in lung diseases—Findings from case reports

Among the case reports published in 2025 in the pulmonary medicine series, some emphasize the range of postoperative and perioperative complications. Despite advancements in surgical and anesthetic techniques, these complications continue to pose significant clinical challenges. The reviewed publications encompass complications directly associated with cardiopulmonary and thoracic surgeries, as well as outcomes of aspiration, ventilation, and endoscopic procedures, which often have atypical or severe courses.

One of the most frequently described complications is postoperative pulmonary edema with varied pathogenesis. Shen et al. reported a severe case of pulmonary edema developing following an interventional procedure for pulmonary hypertension. The authors highlight the role of rapid hemodynamic changes and pulmonary vascular overload, which can lead to rapid respiratory failure requiring intensive treatment.

Another important group consists of aspiration complications, including exogenous lipid pneumopathy, which often develops in the context of medical procedures or swallowing disorders. Li et al. (4) described a case of lipid pneumonia following aspiration of paraffin oil used for therapeutic purposes. This report indicates that the radiological findings may mimic lung cancer or severe pneumonia, which delays an accurate diagnosis. In clinical practice, these cases underscore the need for a detailed perioperative history and for considering lipid aspiration as a cause of atypical parenchymal changes.

Complications related to thoracic surgery and anesthesia techniques are also an important issue. Mao et al. described a pediatric case of pulmonary tuberculoma that was preoperatively interpreted as a benign peripheral tumor. Only during surgery were extensive pleural adhesions identified, which significantly complicated the procedure. This case highlights the risk of underestimating the disease stage based on imaging and the potential surgical consequences of a misdiagnosis. Reports on modern surgical and perioperative techniques highlight the importance of optimizing ventilation and monitoring strategies. Case reports related to VATS procedures performed using the tubeless technique or with advanced monitoring of perfusion and ventilation parameters indicate that even minimally invasive procedures may carry a risk of respiratory destabilization, particularly in patients with limited pulmonary reserve. In this context, case reports serve as early warning signs, enabling the identification of risk factors and the modification of anesthetic protocols.

Recent reports underscore the importance of ventilatory and monitoring strategies in modern surgery. Even minimally invasive procedures like VATS with tubeless techniques or advanced perfusion and ventilation monitoring can pose respiratory risks, especially in patients with limited lung reserve. These case reports serve as warning signs to identify risk factors and adjust anesthetic management. They also highlight that postoperative complications are often multifactorial and require close collaboration among surgical, anesthetic, and pulmonology teams, along with vigilant diagnosis. Data from 2025 reports contribute valuable insights, despite limitations, to improving patient safety and clinical practices. Future research should focus on systematic data collection and integration with surgical registries to better understand the incidence and mechanisms of such complications.

An important aspect of the clinical picture in the cases discussed is the problem of incorrect differential diagnosis between pulmonary infections and neoplastic lesions, as well as the coexistence of these conditions. Zhang et al. described a case of invasive mucinous adenocarcinoma of the lung, whose radiological appearance mimicked pneumonia for a long time, leading to a delay in diagnosis lasting several years and progression of the neoplastic lesion. Similarly, in previously analyzed cases of opportunistic infections, particularly fungal infections, the ability of inflammatory lesions to mimic the radiological appearance of neoplastic lesions was emphasized, which significantly complicates diagnosis. In turn, the publication by Zhu et al., along with its subsequent correction, points to the opposite, equally significant problem—the coexistence of an invasive infection (*Aspergillus fumigatus*) and lung cancer, requiring simultaneous diagnosis and treatment of both conditions. A synthesis of these observations underscores that the distinction between infection and cancer in lung diseases is often clinically blurred, and a delay in diagnosis or failure to account for a coexisting condition can lead to a worsened prognosis. Consequently, it is essential to employ integrated diagnostic strategies that include imaging, histopathological, and microbiological examinations, particularly in cases with an atypical clinical course.

## Conclusion

An analysis of case reports and case series published in 2025 in *Frontiers in Pulmonary Medicine indicates* that rare lung cancers, severe opportunistic infections, and postoperative and perioperative complications remain significant, though often underestimated, causes of mortality in pulmonology. Case reports reveal diagnostic gaps, limitations of standard treatment algorithms, and the potential of new diagnostic and therapeutic technologies to improve patient prognosis.

The conclusions drawn from this review emphasize the need for early, precise diagnosis, close interdisciplinary collaboration, and a willingness to employ non-standard therapeutic strategies in high-risk situations. Case reports, despite their limited evidential value, play a key role in identifying rare but life-threatening clinical scenarios and should be regarded as an important element in improving clinical practice and in designing future systematic reviews.
